# An In Vitro Comparative Analysis of Physico–Mechanical Properties of Commercial and Experimental Bioactive Endodontic Sealers

**DOI:** 10.3390/bioengineering11111079

**Published:** 2024-10-28

**Authors:** Abdulmajeed Kashaf, Faisal Alonaizan, Khalid S. Almulhim, Dana Almohazey, Deemah Abdullah Alotaibi, Sultan Akhtar, Ashwin C. Shetty, Abdul Samad Khan

**Affiliations:** 1 Department of Restorative Dental Sciences, College of Dentistry, Imam Abdulrahman Bin Faisal University, Dammam 31441, Saudi Arabia; 2200600022@iau.edu.sa (A.K.); falonaizan@iau.edu.sa (F.A.); ksalmulhim@iau.edu.sa (K.S.A.); 2 Stem Cell Research Department, Institute for Research & Medical Consultations, Imam Abdulrahman Bin Faisal University, Dammam 31441, Saudi Arabia; daaalmohazey@iau.edu.sa; 3 College of Dentistry, Imam Abdulrahman Bin Faisal University, Dammam 31441, Saudi Arabia; deemahotaibi@gmail.com; 4 Department of Biophysics, Institute for Research & Medical Consultations, Imam Abdulrahman Bin Faisal University, Dammam 31441, Saudi Arabia; suakhtar@iau.edu.sa; 5 Department of Dental Education, College of Dentistry, Imam Abdulrahman Bin Faisal University, Dammam 31441, Saudi Arabia; asyermal@iau.edu.sa

**Keywords:** root canal, sealers, fracture resistance, sealer penetration, bioactive material, confocal laser scanning microscopy

## Abstract

This study aimed to evaluate the fracture resistance of root and sealer penetration after obturation using an epoxy resin sealer AH plus (AH+) and two different bioactive endodontic sealers, i.e., Totalfill BC Hiflow (TF BC), and experimental injectable bioactive glass (Exp.BG). A thermo-sensitive injectable sealer was prepared by using a non-ionic triblock copolymer and bioactive glass. The root canals of human extracted teeth were obturated with the respective sealers. The fracture resistance was analyzed at different time intervals, i.e., days 7, 30, and 90. The morphological and elemental analyses of the fractured roots were conducted with a scanning electron microscopy and a electron dispersive spectroscopy. Sealer penetration depth and the percentage of penetrated sealers into the dentinal tubules were assessed with the confocal laser scanning microscope. Statistical analysis was performed using a one-way ANOVA post hoc Tukey’s test. The mean fracture force in AH+ was significantly higher on day 30 (664.08 ± 138.8 N) compared to day 7 (476.07 ± 173.2 N) and day 90 (493.38 ± 120.18 N). There was no statistically significant difference between the TF BC and Exp.BG at different time intervals. The maximum penetration was observed in the middle region compared to coronal and apical for the Exp.BG, followed by the TF BC and AH+ groups; however, a nonsignificant difference in penetration was found over time. It is concluded that the TF BC group showed overall better fracture resistance than AH+ at day 90. Exp.BG showed comparable sealer penetration to those of TF BC and better than those of AH+.

## 1. Introduction

Endodontic treatment aims to eradicate infection causes and obturate the canal space to prevent further reinfection [1]. However, achieving that goal is accompanied by compromising the structural integrity of the coronal and radicular tooth structure as well as the long-term prognosis of the tooth [2]. Extensive reduction in the radicular dentin during root canal treatment affects the long-term mechanical properties of the tooth [3]. It is established that the overzealous removal of the tooth structure during the root canal treatment contributed to an increased incidence of tooth fracture. Furthermore, dentin dehydration after endodontic therapy and excessive pressure during obturation can lead to root fracture and tooth loss [4].

In addition to conservative preparation, efforts have been made to reinforce the remaining tooth structure using different intracanal sealers and obturation materials [5]. Obturating materials, including sealers, should adhere to the root canal wall consistently and show flexibility similar to the elasticity modulus of dentin to increase fracture resistance [6]. Developing an essential monoblock inside the root canal to coordinate with the elastic modulus of the radicular dentin has been demonstrated to diminish the stresses inside the tooth structure [7]. Tay and Pashley [8] classified the monoblocks within the root canal spaces as primary, secondary, or tertiary depending on the number of interfaces present between the bonding substrate and the bulk core material. According to Belli et al. [9], creating a primary monoblock within the root canal either by an endodontic material or with a post-core system can save the remaining tooth structure or prevent root fractures. It is widely believed that some endodontic sealers can bond to the dentin of root canal and increase the fracture resistance of the endodontically treated teeth [10].

Recently, bioceramics have evolved the endodontic practice due to their widely accepted mechanical and biological properties. Atmeh et al. [11] reported a mineral infiltration zone at the calcium silicate-based material-to-tooth interface, creating a chemical connection. One of the key benefits of calcium silicates is their capacity to generate hydroxyapatite as it sets, which facilitates the formation of a bond between the sealer and the dentinal wall [12]. Bioactive glass (BG) has attracted the interests of many researchers because BG’s ionic dissolution products were found to be osteoinductive. BG is a biocompatible material that can be applied to hard tissues, such as dentin, and soft tissues, such as dental pulp and apical tissue [13]. The BG particles have been incorporated into endodontic sealers to benefit from this material [14]. Two commercially available root canal sealers containing BG are GuttaFlow Bioseal (Coltène/Whaledent AG, Altstetten, Switzerland) and Nishika Canal Sealer BG (CS-BG). Injectable forms of BG are becoming attractive because of their minimally invasive and easy delivery, and have gained interest in biomedical and dental applications [15]. When synthesizing a new endodontic sealer utilizing bioactive materials, careful consideration must be given to their physicochemical and bioactive properties [16].

The clinical desirability of root canal sealers lies in their ability to penetrate the dentinal tubules effectively. It is ideal for these materials to exhibit reliable strength and penetration depth for optimal clinical outcomes [17]. Endodontic sealers and the filling core material have been tested to assess their performance in restoring missing radicular dentin and their sealing ability, as improving these attributes can positively affect the long-term success of treatment [18]. However, with the introduction of new commercialized materials based on bioceramics and the development of experimental materials, it is important to do a comparative analysis with the gold standard epoxy resin sealer. Therefore, this study evaluated the fracture resistance of roots and sealer penetration after obturation using epoxy resin-based and bioactive materials-based sealers. It was hypothesized (H1) that (i) there would be variations in the roots fracture resistance force strength after using epoxy-based and bioactive sealers and (ii) bioactive sealers would show more dentinal tubule penetration compared to epoxy resin sealers. The null hypothesis (H0) was tested against the alternative hypotheses.

## 2. Materials and Methods

Before starting the experimental work, ethical approval was obtained from the institutional review board (#IRB-PGS-2022-02-362). Two commercially available sealers, i.e., AH plus (AH+) and Totalfill BC Hiflow (TF BC), were purchased. Our group prepared the experimental injectable bioactive glass sealer (Exp.BG) as described previously [19]. In short, 10 wt.% non-ionic triblock copolymer pluronic F127 (Poloxamer 407, Sigma Aldrich, St. Louis, MO, USA) was mixed with 24 wt.% hydroxypropyl methylcellulose (Sigma Aldrich, St. Louis, MO, USA). The BG was prepared using the base-catalyzed sol–gel method, and 40 wt.% powder was mixed with 10 mL phosphate-buffer solution and stirred for 2 h. The slurry was mixed with the copolymer and stirred for 3 h at 4 °C. The description of the commercial and experimental materials is given in Table 1.

The sample size calculation was conducted using the formula at a level of significance 0.05 and the power of sample 0.80. The calculated fracture resistance and sealer penetration samples were 90 and 18, respectively.

### 2.1. Preparation of Teeth

A total of 128 sound single-rooted and single canals human extracted teeth were collected and thoroughly cleaned, disinfected with 70/30 ethanol solution for 10 min, and stored in 0.5% thymol solution (not more than two months). All disinfected teeth were examined under a light microscope to exclude teeth cracks or open apices. All teeth were decoronated using Isomet 5000 BUEHLER, Lake Bluss, IL, USA, to achieve a standardized length of 13 mm. The buccolingual and mesiodistal diameters of the coronal planes were measured by using a digital caliper (UNIOR Products, Zreče, Slovenia/EU). All the roots were of similar dimensions, measuring 6.5 ± 0.4 mm buccolingually and 5.0 ± 0.5 mm mesiodistally. The teeth were randomly distributed into three groups. The group distribution was carried out according to the sealer’s application: (i) G1: AH+ sealer and gutta-percha, (ii) G2: TF BC sealer and gutta-percha, and (iii) G3: Exp.BG and gutta-percha.

Each canal was accessed, and the working length was determined by subtracting 1 mm from the length of an inserted #10 K-file with its tip visualized at the apical foramen. The canals were instrumented using Vortex rotary files (Dentsply Sirona, Bensheim, Germany) up to a master apical file size #40 (0.04). A 3 mL of 2.5% sodium hypochlorite (NaOCl) irrigation between each file size was conducted using a 27-G syringe. After completion of the canal preparation, a final rinse was performed with 2 mL 2.5% NaOCl followed by ultrasonic activation for two consecutive 30 s cycles (total of 1 min) followed by 2 mL 17% EDTA and activation for 1 min using ultrasonic handpiece (Ultra X Eighteenth) with tip S21 SIZE 20/02 worked at the maximum power of 45 kHz. Then, each canal was washed with saline and dried with sterile paper points. After instrumentation, all the roots were obturated using single cone gutta-percha size 0.04 taper with the corresponding sealer type for each group. After completing the obturation, periapical radiographs were taken to assess the quality of the root filling. The coronal cut surface and the apical foramen were coated with nail varnish. All teeth were stored at 37 °C and 100% humidity before being subjected to the test.

### 2.2. Fracture Resistance Test

For the fracture resistance test, 90 teeth were divided into each group (n = 30). Each group was subdivided into three subgroups (n = 10) according to the storage time, i.e., 7, 30, and 90 days. Another 20 roots were used as a control, where 10 roots were not prepared (negative control), and the other 10 were prepared without filling (positive control). The values of positive and negative control groups were taken on day 7. The apical 5 mm root surfaces were coated with a thin layer of 0.2 to 0.3 mm polyvinylsiloxane impression material (Dentsply Aquasil, Milford, DE, USA) to simulate a periodontal membrane using a micro brush. The roots were mounted individually and vertically in a self-cure acrylic resin, exposing 8 mm of the coronal parts of the roots. A universal testing machine (Instron Model 5965, Norwood, MA, USA) was used, whereby a stainless-steel jig with a tip of 3 mm in diameter was used to apply vertical force over the canal orifice. The crosshead speed was 1 mm·min^−1^, and a slowly increasing vertical force was exerted until fracture occurred. The fracture moment was determined when a sudden drop in force occurred. The maximum force required to fracture each specimen was recorded in newtons. The fractured roots were gold coated using gold sputter (Quorum Technology, Lewes, UK) for 90 s, and the surfaces were examined using a scanning electron microscope equipped with energy dispersive spectroscopy EDS (TESCAN VEGA 3, TESCAN, Brno, Czech Republic).

### 2.3. Sealer Penetration Test

A total of six roots of each group were instrumented and obturated using the same technique as in the previous two tests with the same three different sealers. Before obturation, sealers were mixed with 0.1% fluorescein to allow for confocal laser scanning microscopy analysis. The fluorescein-sealer mixture was delivered into the canal using the gutta-percha cone.

#### Confocal Laser Scanning Microscopic (CLSM) Analysis

After the final obturation, samples were stored at 37 °C and 100% relative humidity to permit the complete set of sealers. Samples were embedded into resin blocks and were sectioned transversely at 2, 5, and 8 mm from the apex to represent the apical, middle, and coronal thirds, respectively. The slices were photographed on days 7, 30, and 90 under a CLSM (Zeiss LSM 510; Carl Zeiss, Jena, Germany) and an epifluorescence method with wavelengths of absorption and emission for fluorescein of 536/617 nm. The images were analyzed using ImageJ software, version 1.54h to measure the penetration depth of the sealer and the percentage of penetrated sealers into the dentinal tubules.

### 2.4. Statistical Analysis

All the data were collected and analyzed using SPSS version 23 (IBM Software, Armonk, NY, USA). Means and standard deviations were calculated. A one-way ANOVA with a post hoc Tukey’s test was used to compare the groups. A *p*-value of < 0.05 was considered statistically significant.

## 3. Results

### 3.1. Fracture Resistance Test

The mean and standard deviation values of the fractured roots are presented in Table 2. On day 7, a nonsignificant difference (*p* = 0.064) was found between the groups. The TF BC sealer observed the highest mean force (627.46 ± 188.14 N), whereas the weakest force required was observed in the positive control group (457.67 ± 109.7 N). The mean force required to fracture the roots of the negative control was (507.9 ± 104.5 N). On day 30, the highest mean force was observed for the AH+ group (664.08 ± 138.8 N), and a significant difference was found with the negative control group (*p* = 0.040), positive control group (*p* = 0.003), and the Exp.BG group (*p* = 0.031). However, no significant difference was found between the AH+ and TF BC sealer groups (*p* = 0.683). On day 90, the maximum mean force required to fracture the roots was observed in the Exp.BG group, whereas no significant differences were found between the groups (*p* = 0.124).

For the comparison within each sealer at different time intervals, the mean fracture force in AH+ was significantly higher at day 30 compared to day 7 (*p* = 0.020) and day 90 (*p* = 0.037). There was no statistically significant difference in the mean fracture force within the groups of TF BC (*p* = 0.803) and Exp.BG (*p* = 0.165) at different time intervals.

**Figure 1:**
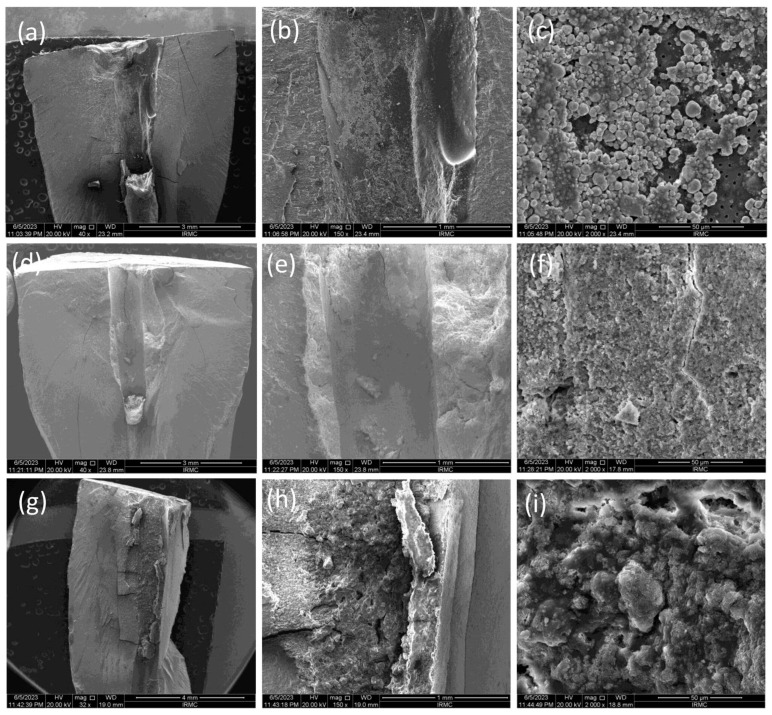
SEM images on day 7, where images were taken at 40×, 150×, and 2000×. The images showed the presence of remanent sealers, i.e., (**a**–**c**) AH+, (**d**–**f**) TF BC, and (**g**–**i**) Exp.BG.

**Figure 2:**
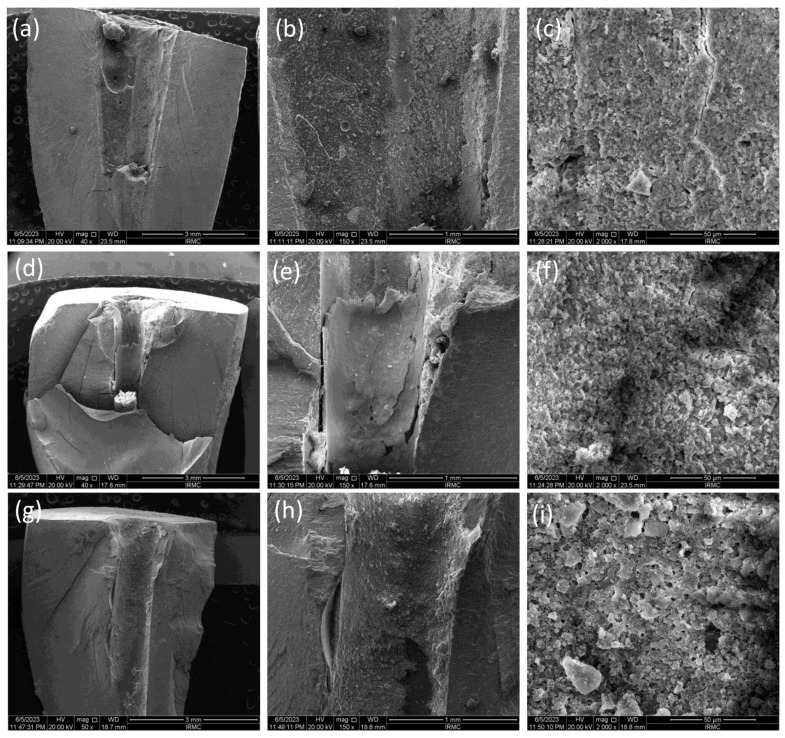
SEM images at day 30, where images were taken at 40×, 150×, and 2000×. The images showed the presence of (**a**–**c**) AH+, (**d**–**f**) TF BC, and (**g**–**i**) Exp.BG sealers on the root dentin.

**Figure 3:**
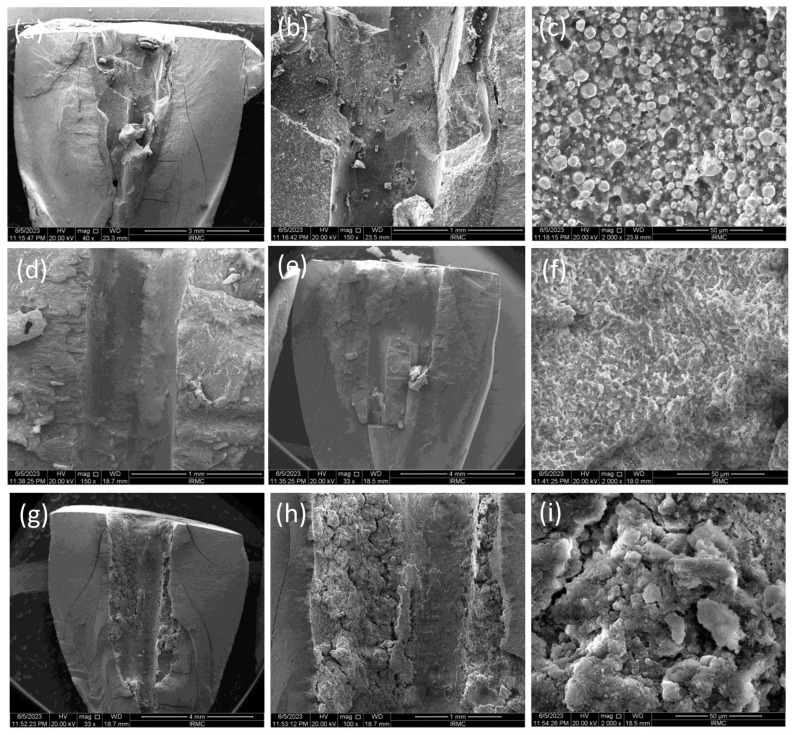
SEM images at day 90, where images were taken at 40×, 150×, and 2000×. The images showed the presence of (**a**–**c**) AH+, (**d**–**f**) TF BC, and (**g**–**i**) Exp.BG sealers on root dentin.

The SEM images (Figure 1, Figure 2 and Figure 3) showed the fractured surfaces of the roots at different magnifications after the fracture resistance test at different time intervals. The root surfaces showed the presence of sealers, whereas, on day 7 (Figure 1a–c), the AH+ showed uniform distribution of the sealer, and a similar trend was observed for the Exp.BG (Figure 1g–i). However, TF BC showed non-uniform behavior (Figure 1d–f). A different behavior was observed on day 30 (Figure 2a–i), where TF BC and Exp.BG showed a layer of remnants of the sealer, whereas the AH+ showed a discrete presence of sealers on root dentin. At day 90 (Figure 3a–i), a thick layer of sealers was found on the TF BC and Exp.BG groups. The layers covered the entire surface of the root dentin. The AH+ showed almost the same behavior as on day 30.

**Figure 4:**
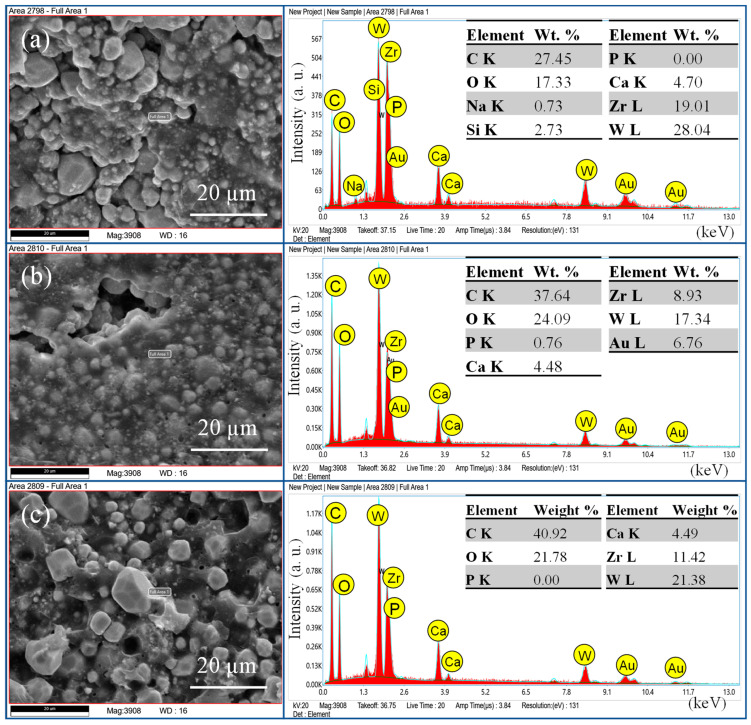
EDX pattern of AH+ at (**a**) day 7, (**b**) day 30, and (**c**) day 90.

**Figure 5:**
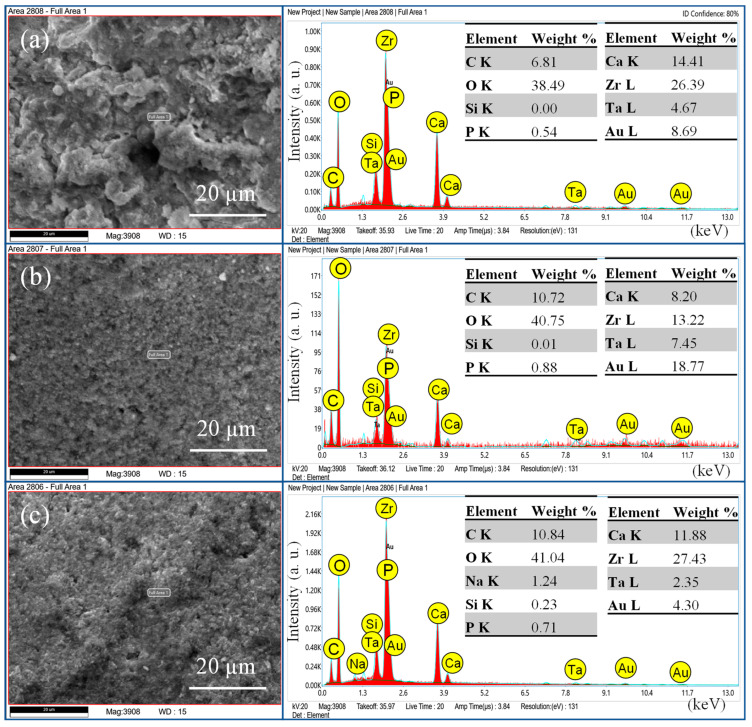
EDX pattern of TF BC Hiflow at (**a**) day 7, (**b**) day 30, and (**c**) day 90.

**Figure 6:**
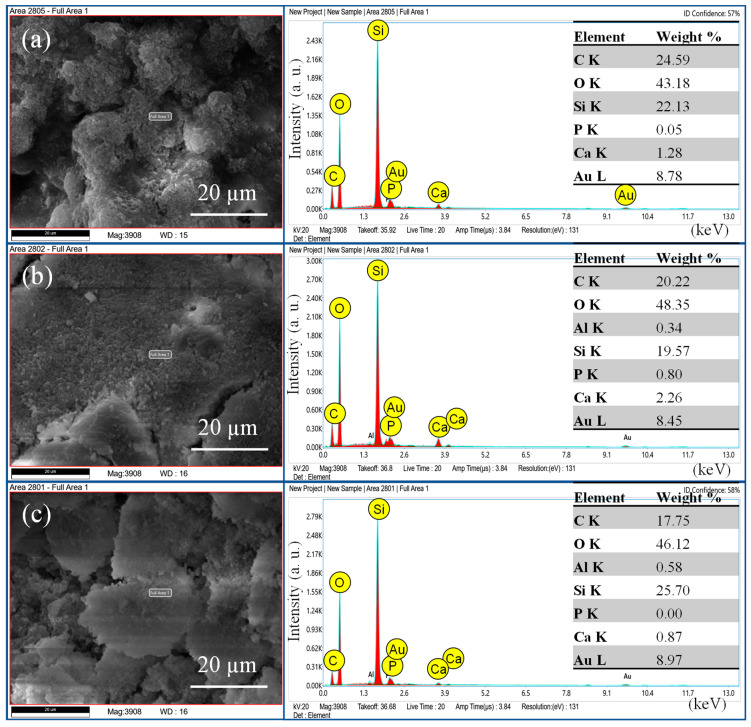
EDX pattern of Exp.BG at (**a**) day 7, (**b**) day 30, and (**c**) day 90.

The EDX pattern (Figure 4, Figure 5 and Figure 6) confirmed the presence of elements in respective sealers, where AH+ showed (Figure 4) the presence of zirconium, tungsten, and calcium. The weight percentage of calcium was almost the same from day 7 to 90. The silicon appeared only on day 7 samples but was not present on day 30 and 90 samples. There was no presence of phosphates in this group. The TF BC group also showed the presence of calcium along with zirconium and tantalum. Interestingly, the silicon did (Figure 5) not appear in this group on days 7, 30, and 90. The Exp.BG group showed (Figure 6) the maximum presence of silicon along with calcium. Minor amounts of phosphate were also present in this group.

### 3.2. Sealer Penetration Analysis

The mean penetration values of all sealers at three-time intervals are tabulated in Table 3. It was found that the maximum penetration was observed for the Exp.BG, followed by the TF BC and AH+ groups; however, not much difference in penetration was found with time.

**Figure 7:**
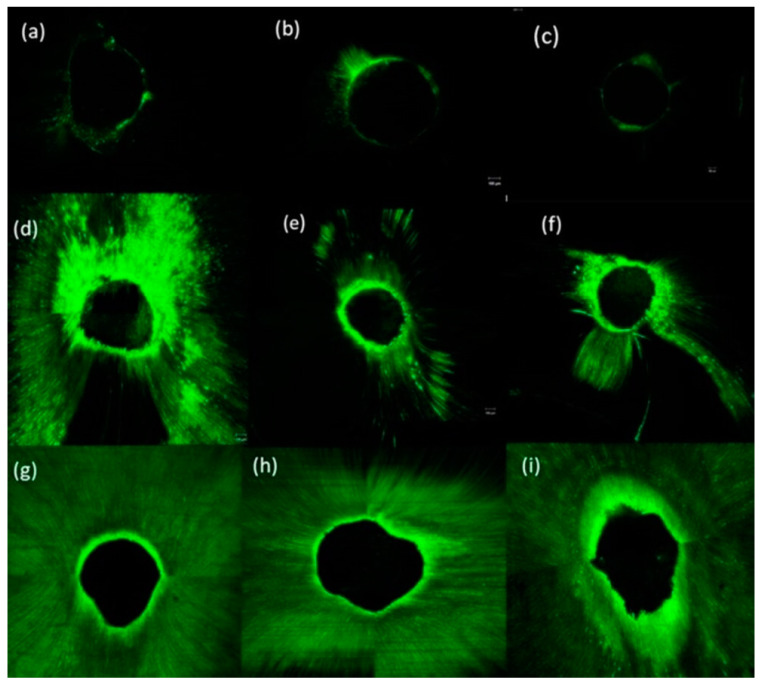
The representative images of CLSM showing the penetration of sealers at day 30: (**a**–**c**) coronal, middle, and apical of AH+; (**d**–**f**) coronal, middle, and apical of TF BC, and (**g**–**i**) coronal, middle, and apical of Exp.BG.

**Figure 8:**
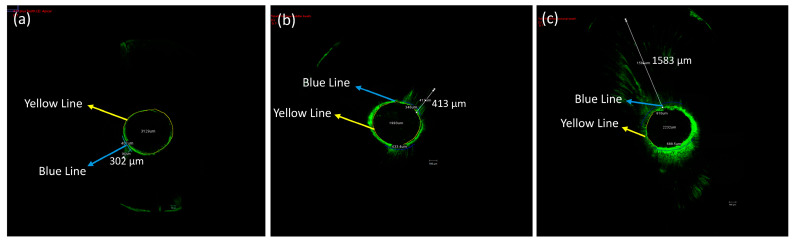
The representative images of (**a**) AH+, (**b**) TF BC, and (**c**) Exp.BG showing the method of calculating the deepest penetration and the penetration percentage. The penetration area (blue lines) was divided by the canal circumference (yellow line). The representative images show the penetration depth (white arrow) of each group.

Concerning the percentage of sealer penetration to the canal circumference, the mean percentage of AH+ sealer penetration in the coronal section at days 7, 30, and 90 was 12.26%, 13.87%, 12.16%; in the middle: 22.72%, 26.89%, 21.67%; in the apical: 27.42%, 27.7%, 27.17%, respectively. In the TF BC, the mean percentage of penetration in the coronal section on days 7, 30, and 90 was 78.36%, 75.31%, 77.87%; in the middle: 61.88%, 62.35%, 62.99%; in the apical: 68.90%, 66.85%, 74.62%, respectively. In comparison, the mean percentage of the Exp.BG penetration in the coronal section at days 7, 30, and 90 was 98.97%, 99.62%, 97.83%; in the middle: 100%, 99.63%, 99.42%; in the apical: 92.38%, 92.26%, 91.51%, respectively. The representative images of CLSM of three sealers at the coronal, middle, and apical regions are given in Figure 7 and Figure 8.

## 4. Discussion

An endodontic material should exhibit adequate mechanical properties and sealing ability. Many new endodontic sealers based on calcium silicates and bioactive glass have recently been developed and marketed [20]. However, their mechanical properties and penetration depth performances are still unclear. This study achieved the objectives, and a comparative study was conducted to evaluate the mechanical and physical properties of epoxy-based and bioactive materials-based endodontic sealers.

It is advisable to standardize all controllable factors when utilizing extracted human teeth for tests [21]. Even so, the potential for uncontrollable variations still exists. In this study, a single operator performed the standardized procedure; however, variations in root canal shapes and morphologies are anticipated in human extracted teeth. Therefore, high standard deviation values were observed. The primary focus of this experimental study was to evaluate the impact of time on the mechanical and physical properties of root-treated teeth. The main reason for including radicular tooth structures in this study was that they are less resistant to fracture after endodontic treatment due to loss of dentin during instrumentation and dehydration. The fracture resistance test attempted to minimize the loss of dentin by using less taper files (0.04 taper) to avoid significant and unpredictable differences in strength. The results of this study showed no significant difference in the mean fracture force between non-instrumented and instrumented roots without filling, which might be due to the minimal radicular dentin removal. Furthermore, all the filled root groups showed a higher mean fracture resistance force than instrumented roots without filling on day 7; however, the difference was nonsignificant. It is important for clinicians to ensure that the outcomes of the instrumentation and obturated roots are comparable to those of the intact roots.

The present study showed increased fracture resistance in TF BC and the Exp.BG groups were observed at all time intervals of the test and compared to non-prepared intact roots, whereby the values were lower in AH+ groups at days 7 and 90. The high fracture resistance of the bioactive materials-based groups could be due to a better distribution of the injectable sealers on the dentin wall. The SEM images confirmed the presence of more bioactive materials-based sealers than the AH+. It was also observed that the layer was well-distributed on the root dentin surface with time. It is assumed that the gaps between the walls of the root canal and gutta-percha cones were filled by the bioactive materials-based sealer, representing the main intended function of a sealer. On day 30, the AH+ group showed a higher value than other sealers. However, the difference was nonsignificant, whereas the difference was significant in the positive and negative control groups. The results of this present study are comparable to the previous study [22], where the investigators evaluated the time factor on fracture resistance using different sealers and found higher values for epoxy resin sealer after one month compared to the first week. Bioactive materials-based root canal sealers tend to make a chemical bonding to the walls of the root canal dentin [23]. Similar behavior was observed in this study, where bonding of the bioactive materials-based sealer was improved with time, consequently improving the fracture resistance of obturated teeth; however, the difference was nonsignificant. It is important to know the ion-nucleation theory to better understand the overall high values of bioactive materials-based sealers. It is expected that the calcium and phosphate ions were released from the material and deposited on the root dentin, which could influence the physico–mechanical properties of the materials. However, once the released ions convert into crystals and eventually form the apatite layer, they can gain their physico–mechanical properties.

Another possible reason for non-linear behavior in the results could be related to the variations in teeth. The age and accurate condition of the root dentin are unknown. The obliteration of the dentinal tubules and the inner structure might influence the result. In this experiment, 2.5% NaOCl was activated for one minute, followed by the activation of EDTA for 1 min using ultrasonics. Kuah et al. [24] showed that a 1 min application of EDTA with ultrasonics efficiently removes smear and debris at the apical region of the instrumented root canal. It is reported that a high concentration of NaOCl caused significant changes to the fracture strength of root dentine [25]. Furthermore, a higher concentration of NaOCl could negatively influence the bond of epoxy resin to dentin by removing the collagen network and producing an oxygen layer, inhibiting the resin sealer’s polymerization [26]. Therefore, in this experiment, 2.5% NaOCl was used as an irrigant to minimize such destruction.

This study tested the penetration only in the single cone obturation technique. It is suggested that different root canal filling techniques promoted similar penetration of the root canal sealer into dentinal tubules [27]. The penetration depth depends on the particle size of the fillers in sealers. The particle size of fillers in TF BC and Exp.BG was approx. 0.2 μm in size on average. This characteristic might facilitate better dentin tubule penetration. The SEM images of the particle size and the CLSM images confirmed this claim. This study demonstrated a noticeably better performance of TF BC and Exp.BG penetration compared to AH+ was evident. In addition to that, SEM images showed a larger particle size of AH+ sealer, which might have shown a lower penetration ability. Based on these results, it is assumed that better sealer penetration, uniform distribution, and formation of the new apatite layer can improve the fracture resistance.

## 5. Conclusions

Within the limitations of this in vitro study, it is concluded that the null hypothesis was rejected. The TF BC and Exp.BG groups showed comparable fracture resistance and overall better than AH+ on day 90. Overall, TF BC sealer exhibited better physico–mechanical properties than AH+. Exp.BG showed maximum penetration of the dentin. The fracture resistance and the sealer’s penetration were found to be not time dependent. The flow characteristics of sealers, the particle size of sealers, irrigation techniques, and the number of dentinal tubules is all major factors in penetration depth and percentage of penetration.

## Figures and Tables

**Table 1:** Composition of the commercial and experimental materials tested.

Groups	Composition	Manufacturer
Total Fill BC Sealer Hiflow	Zirconium oxide, Tricalcium silicate, Dicalcium silicate, calcium hydroxide	FKG Dentaire SA Lashaudfon, Switzerland
AH plus	Bisphenol-A and F epoxy resin, calcium tungstate, zirconium oxide, silica, iron oxide dibenzyl diamine, aminoadamantane, tricyclodecane diamine, silicone oil.	Dentsply Sirona, Bensheim, Germany
Injectable Bioactive Glass	SiO_2_, CaO, Na_2_O, P_2_O_5_, F127 (Poloxamer 407), hydroxypropyl methylcellulose	Experimental ^1^ [19]

^1.^Experimental Injectable BG was prepared at the Interdiscipllinary Research Centre in Biomedical Materials, COMSATS University Islamabad, Lahore Campus, Pakistan.

**Table 2:** Mean and standard deviation of fracture resistance test in Newtons of +ve control, -ve control, commercial and experimental sealers-based groups.

Days	+ve Control	−ve Control	AH +	TF BC	Exp.BG	*p*-Value
Day 7	457.67 ± 109.7	507.9 ± 104.5	476.07 ± 173.2	627.46 ± 188.14	517.93 ± 51.3	0.064
Day 30			664.08 ± 138.8	594.17 ± 149.9	502.64 ± 76.23	0.002
Day 90			493.38 ± 120.18	580.12 ± 149.74	581.26 ± 136.41	0.124
*p*-value			0.013	0.803	0.165	

**Table 3:** The sealer penetration depth (µm) of commercial and experimental sealers at days 7, 30, and 90.

Sections	Days	AH+	TF BC	Exp.BG
Coronal Section	Day 7	347.6 ± 212	1362.5 ± 309	1583 ± 390
	Day 30	311 ± 183	1366 ± 186	1653.5 ± 348
	Day 90	341 ± 278	1363 ± 281	1532.5 ± 461
Middle Section	Day 7	411 ± 167	821.5 ± 301	1901 ± 304
	Day 30	368.5 ± 122	817.5 ± 321	1902.5 ± 306
	Day 90	393.5 ± 191	502.04 ± 358	1905 ± 309
Apical Section	Day 7	294.7 ± 92	613 ± 185	1118 ± 251
	Day 30	280.5 ± 101	566 ± 131	1110 ± 250
	Day 90	295.2 ± 99	608.5 ± 184.5	1104 ± 240

## Data Availability

Data are available on request.
